# Prescription for Psoriasis

**Published:** 1890-03

**Authors:** 


PRESCRIPTION FOR PSORIASIS.—The favorite prescription of Mr. Jonathan Hutchinson for psoriasis is:

℞—Acid chrysophanic, . . . . . . . . . . . . . . . . . grains x.
Liq. carbonis deterg. (Wright's), . . . . . . . . . . m x.
Hydr. amm. chlorid., . . . . . . . . . . . . . . . . . grains x.
Adip. benzoat., . . . . . . . . . . . . . . . . . . . ℥ j.
Misce, fiat unguent.

At night the patient should wash the diseased surfaces free from all scales; then, standing before a fire, rub on the ointment, devoting, if possible, half an hour to the operation. The proportion of chrysophanic acid is not irritating, and stains the linen but slightly. With some cases, even a weaker chrysophanic ointment is entirely sufficient. Internally, Mr. Hutchinson prescribes arsenic, though he is not convinced that it is an important adjunct.—*Archives of Surgery*, July, 1889.

